# Clinical characteristics analysis of pertussis and Mycoplasma pneumoniae infection in children

**DOI:** 10.3389/fcimb.2026.1711371

**Published:** 2026-02-27

**Authors:** Min Xue, Xiaoling Wei, Bing Wang, Miao Liu, Yun Zhang, Xiang Ma

**Affiliations:** 1 Jinan Institute of Pediatric Research, Children’s Hospital Affiliated to Shandong University (Ji’nan Children’s Hospital), Jinan, Shandong, China; 2 Jinan Key Lab of Respiratory Diseases for Children, Jinan Children’s Hospital, Jinan, Shandong, China; 3 Shandong Provincial Clinical Research Center for Children’s Health and Disease, Jinan Children’s Hospital, Jinan, Shandong, China; 4 Department of Health Data Application and Management, Children’s Hospital Affiliated to Shandong University(Ji’nan Children’s Hospital), Jinan, Shandong, China; 5 Department of Respiratory Medicine, Children’s Hospital Affiliated to Shandong University (Ji’nan Children’s Hospital), Jinan, Shandong, China

**Keywords:** children, clinical characteristics, infection, mycoplasma pneumoniae, pertussis, positivity

## Abstract

**Objective:**

To analyze the clinical differences between pertussis and/or *Mycoplasma pneumoniae* (MP) infections in children and provide insights for clinical differential diagnosis.

**Methods:**

We retrospectively reviewed children with respiratory symptoms who attended Shandong University Children’s Hospital (Jinan, China) from 2019 to 2024 and underwent simultaneous testing for pertussis and MP. Patients were categorized as pertussis-only, MP-only, or dual-positive, and differences in demographics, seasonality, manifestations, hematologic indices, and co-detected pathogens were analyzed.

**Results:**

A total of 7184 children were included: 2,982 pertussis-only, 3,166 MP-only, and 1,036 dual-positive. Significant differences were observed in sex (χ² = 30.964), age (χ² = 393.010), and season (χ² = 436.070) (all *p* < 0.001). Pertussis-only cases were more common in boys, during spring and winter, and in patients aged 6 years to <12 years. MP-only cases clustered in ages 2 to <6 and 6 to <12 years, with peaks in summer and autumn. Dual-positive cases were slightly more frequent in girls, clustered in the 6 to <12-year-old group, and occurred more often in spring and summer. Fever (χ² = 442.36, *p* < 0.001) was more frequent in the MP-only and dual-positive groups, whereas gastrointestinal symptoms (χ² = 30.00, *p* < 0.001), cyanosis (χ² = 12.91, *p* = 0.002), spasmodic cough (χ² = 212.07, *p* < 0.001), and cockcrow-like echo (χ² = 77.38, *p* < 0.001) were more common in the pertussis-only group. Lung crackles (χ² = 52.44, *p* < 0.001) and multilobar involvement (χ² = 28.08, *p* < 0.001) were predominantly observed in the MP-only group. The duration of cough before diagnosis was shorter in the MP-only group than in both the pertussis-only and dual-positive groups (H = 371.49, *p* < 0.001). Lymphocyte counts (H = 178.03) were the highest in the pertussis-only group, and neutrophil counts (H = 119.45) and C-reactive protein (H = 369.80) were the highest in the MP-only group (all p<0.001). Among the 7184 children, 1,224 (15.65%) had codetection of other pathogens, with human rhinovirus, *Haemophilus influenzae*, and *Streptococcus pneumoniae* most common. The MP-only group was more often accompanied with influenza A/B (χ² = 16.688, *p* < 0.001) and *Legionella pneumophila* (χ² = 12.715, *p* = 0.002); pertussis-only, *Streptococcus pneumoniae* (χ² = 11.872, *p* = 0.003); dual-positive, *Klebsiella pneumoniae* (χ² = 7.284, *p* = 0.009).

**Conclusion:**

Pertussis and MP infections in children show distinct demographic, seasonal, clinical, and laboratory patterns. Recognition of these epidemiologic and clinical signatures supports early differentiation at the bedside and the use of multiplex PCR combined with specific laboratory markers to enable more targeted clinical management.

## 1 Introduction

Pertussis, caused by *Bordetella pertussis*, is an acute respiratory infection marked by persistent, paroxysmal coughing, with a typical course lasting two to three months and even longer in severe cases. Although it can affect individuals of all ages, infants are particularly vulnerable, and pertussis remains a leading cause of infant mortality worldwide (Decker and Edwards, 2021). Infants who contract pertussis often experience severe complications, such as apnea, asphyxia, and airway obstruction, which can lead to hypoxemia and other life-threatening conditions (Decker and Edwards, 2021). The widespread introduction of pertussis vaccines in the second half of the 20th century led to a marked global decline in incidence. However, many countries have since experienced a resurgence, drawing renewed international attention (Cherry, 2012). Epidemiological studies suggest that this resurgence is accompanied by shifts in susceptibility, with the age of onset gradually moving upward in children (MacIntyre et al., 2024). Similar trends have been observed in China, with rising incidence and a broadening of the affected population from infants to school-age children and adolescents (Liu et al., 2024). In school-age children and adolescents, although complications are relatively fewer, they often experience persistent severe coughing, wheezing, apnea, and sleep disturbance (Decker and Edwards, 2021). At the same time, the increasing prevalence of macrolide-resistant *B. pertussis* strains has complicated treatment, and associated mortality has risen (Liu et al., 2024).

*Mycoplasma pneumoniae* (MP) is a major atypical respiratory pathogen in children. It circulates worldwide across all age groups but is most frequently identified in preschool children and adolescents, with the incidence typically peaking in autumn and winter (Yan et al., 2024; Diaz et al., 2025). MP infection often presents with paroxysmal dry cough, which is occasionally accompanied with wheeze. Its symptoms overlap with those of pertussis and can produce a “pertussis-like” syndrome. In school-age children and adolescents, MP infection is often associated with a prolonged disease course and chronic respiratory issues. These patients are prone to recurrent coughing, expectoration, and even hemoptysis as complications (Wang et al., 2024). In recent years, broader access to diagnostic testing has driven fluctuating increases in MP detection, while macrolide-resistant strains have risen steadily. In some regions of China, macrolide resistance now exceeds 80%, which poses a substantial therapeutic challenge (Chen et al., 2024; Xu et al., 2024).

Pertussis and MP infection are common pediatric conditions with overlapping features, including paroxysmal cough, wheeze, and fever, which make symptom-based diagnosis difficult. Age was once a key clue, but recent shifts in epidemiology and clinical profiles have reduced its value. The spread of molecular diagnostics has enabled earlier identification, which highlights the need to reassess clinical patterns. Dual-positive detections of pertussis and MP infection are increasing. However, few studies have compared demographic, seasonal, and laboratory profiles of pertussis, MP, and co-infection in children, especially in the context of changing epidemiology post-pandemic. To improve differential diagnosis and early recognition, we retrospectively analyzed cases of pertussis and/or MP infection at Shandong University Children’s Hospital (Jinan, China) from 2019 to 2024 and compared the epidemiology, clinical manifestations, and laboratory findings.

## 2 Methods

### 2.1 Study population

#### 2.1.1 Study sample

We retrospectively included children aged 0–18 years who presented to Shandong University Children’s Hospital with respiratory symptoms (cough, fever, or dyspnea) between 2019 and 2024. All eligible patients underwent nucleic acid testing for both *Bordetella pertussis* and MP.

#### 2.1.2 Inclusion, exclusion, and grouping criteria

The inclusion criteria were as follows: ① aged 0–18 years; ② presence of respiratory symptoms; ③ pertussis and MP PCR assays performed on samples collected on the same day. ④ availability of complete laboratory results and clinical records.

The exclusion criteria comprised the following: ① absence of pertussis and MP PCR assays performed on samples collected on the same day. ② incomplete laboratory or clinical data.

The grouping criteria consisted of the following: by pathogen: pertussis-positive only, MP-positive only, and dual positive;

By age: <3 months, 3 months to <2 years, and 2 to <6, 6 to <12, and ≥12 years; by season: spring (March–May), summer (June–August), autumn (September–November), and winter (December–February).

#### 2.1.3 Study variables

The demographics included the following: sex, age, and season of presentation. Clinical manifestations were as follows: wheeze or shortness of breath, fever, gastrointestinal symptoms (vomiting, diarrhea, abdominal pain, or poor appetite), cyanosis, dyspnea, lung crackles, and multilobar involvement. Laboratory findings included the following: complete blood count (lymphocyte, neutrophil, white blood cell, and platelet counts), C-reactive protein, and results of additional pathogen testing (viral, bacterial, and atypical pathogens).

### 2.2 Laboratory assays

Nucleic acid detection for pertussis and MP infection was performed using a polymerase chain reaction (PCR) fluorescence probe assay. The experiment was conducted strictly following the kit instructions, with a cycle threshold value 38 considered positive in accordance with the manufacturer’s protocol. (MA-6000, Suzhou Yarui Biotechnology Co., Ltd.; MP/*Bordetella pertussis* nucleic acid detection kit, Beijing ZC-HS Biotechnology Co., Ltd.). Complete blood counts were obtained using an automated hematology analyzer and included parameters such as total white blood cell and lymphocyte counts and percentages. Other respiratory viruses were identified via PCR fluorescence probe assays and bacterial pathogens through multiplex fluorescent PCR.

### 2.3 Statistical analysis

All statistical analyses were conducted using R software (version 4.0.3). Categorical variables were summarized as frequencies and percentages and compared using chi-square test or Fisher’s exact test, as appropriate. Continuous variables were expressed as medians with interquartile ranges (IQRs) and compared across groups using the Kruskal–Wallis test with Bonferroni correction was exclusively applied to *post-hoc* pairwise Mann−Whitney U tests following a significant omnibus Kruskal–Wallis test, with the significance threshold adjusted for the number of pairwise contrasts. Missing data were handled by listwise deletion. A two-sided *p*- value <0.05 was considered statistically significant.

## 3 Results

### 3.1 Baseline characteristics

A total of 14,981 children underwent dual testing for the detection of pertussis and MP infection. After the exclusion of 7,797 with negative results for both pathogens or those with incomplete records, 7,184 children were included in this work. Among them, 3,902 (54.3%) were male, and 3,282 (45.7%) were female (male-to-female ratio ≈1:1.19). The ages ranged from 12 days to 17.9 years, with a median of 4.7 years (IQR: 2.4–7.0). Based on the pathogen profile, 2,982 (41.5%), 3,166 (44.1%), and 1,036 (14.4%) belonged to the pertussis-positive only, MP-positive only, and dual-positive groups, respectively. In terms of pertussis vaccination status, out of the total population, 82.90% (2,472) were fully vaccinated, 14.22% (424) received partial vaccination, and 2.88% (86) were unvaccinated.

### 3.2 Sex distribution by positivity pattern

Overall, the sex distribution differed significantly among positivity groups (χ² = 30.964, *p* < 0.001). In the pertussis-only group, 57.6% were male and 42.4% female, whereas in the dual-positive group, 47.9% were male and 52.1% female; both differences showed statistical significance (χ² = 21.215 and 19.928, *p* < 0.001). In addition, within male and female subgroups, the distribution of positivity patterns varied significantly (*p* < 0.05) (**Table 1**).

**Table 1:** Comparison of sex distribution by positivity pattern.

Sex group	Pertussis-positive only	MP-positive only	Dual-positive	Total
Female	1266 (42.45%)^a^	1476 (46.62%)^b^	540 (52.12%)^c^	3282
Male	1716 (57.55%)^a^	1690 (53.38%)^b^	496 (47.88%)^c^	3902
Total	2982	3166	1036	7184
χ2	21.215	1.930	19.928	30.964
*P*	<0.001	0.165	<0.001	<0.001

Superscript letters indicate subsets of positive types; same letters indicate no significant difference, different letters imply a significant difference (P<0.05).

### 3.3 Age distribution by positivity pattern

Overall, the distribution of positivity patterns differed significantly across age groups (χ² = 393.010, *p* < 0.001). Pertussis-only cases clustered in the 6 to <12 years group (50.37%; χ² = 234.070, *p* < 0.001). MP-only cases were concentrated in the 2 to <6 (45.29%) and 6 to <12 years (40.81%) groups (χ² = 306.410, *p* < 0.001). Dual-positive cases were also predominantly 6 to <12 years (61.39%; χ² = 99.007, *p* < 0.001). Pairwise comparisons showed that in the 6 to <12-year-old group, all three positivity patterns varied significantly (*p* < 0.05). In the <3-months, 3 months to <2 years, and ≥12-year groups, pertussis-only differed from the other patterns, whereas MP-only and dual-positive did not differ significantly. In the 2 to <6 years group, pertussis-only differed significantly from MP-only, and dual-positive did not (**Table 2**).

**Table 2:** Comparison of age distribution by positivity pattern.

Age group	Pertussis-positive only	MP-positive only	Dual-positive	Total
<3 months	86 (2.88%)^a^	12 (0.38%)^b^	1 (11.39%)^b^	99
3 months to <2 years	501 (16.80%)^a^	376 (11.88%)^b^	118 (11.39%)^b^	995
2 to <6 years	798 (26.76%)^a^	1434 (45.29%)^b^	263 (25.39%)^a^	2495
6 to <12 years	1502 (50.37%)^a^	1292 (40.81%)^b^	636 (61.39%)^c^	3430
≥12 years	95 (3.19%)^a^	52 (1.64%)^b^	18 (1.74%)^b^	165
Total	2982	3166	1036	7184
χ2	234.07	306.41	99.007	393.01
*p-*value	<0.001	<0.001	<0.001	<0.001

Superscript letters indicate subsets of positive types; same letters indicate no significant difference, different letters imply a significant difference (*p* < 0.05).

### 3.4 Seasonal distribution by positivity pattern

Overall, the distribution of positivity patterns differed significantly across seasons (χ² = 436.070, *p* < 0.001). MP-only cases were most frequent in autumn (32.31%) and summer (30.95%) (χ² = 425.990, *p* < 0.001); pertussis-only cases in spring (33.13%) and winter (27.67%) (χ² = 290.700, *p* < 0.001); dual-positive cases in spring (33.98%) and summer (25.19%) (χ² = 32.471, *p* < 0.001). After Bonferroni correction, pairwise comparisons showed that the composition differed across all three patterns in summer (*p* < 0.05), whereas in the other seasons MP-only and dual-positive did not differ significantly (*p* > 0.05) (**Table 3**).

**Table 3:** Comparison of seasonal distribution by positivity pattern.

Season	Pertussis-positive only	MP-positive only	Dual-positive	Total
Winter	825 (27.67%)^a^	459 (14.50%)^b^	239 (23.07%)^b^	1523
Summer	702 (23.54%)^a^	980 (30.95%)^b^	261 (25.19%)^c^	1943
Spring	988 (33.13%)^a^	704 (22.24%)^b^	352 (33.98%)^b^	2044
Autumn	467 (15.66%)^a^	1023 (32.31%)^b^	184 (17.76%)^b^	1674
Total	2982	3166	1036	7184
χ^2^	290.7	425.99	32.471	436.07
*p-*value	<0.001	<0.001	<0.001	<0.001

Superscript letters indicate subsets of positive types; same letters indicate no significant difference, different letters imply a significant difference (P<0.05).

### 3.5 Distribution of clinical manifestations and hematologic indices by positivity pattern

In terms of clinical manifestations, significant differences were observed across positivity patterns for fever (χ² = 442.36, *p* < 0.001), gastrointestinal symptoms (χ² = 30.00, *p* < 0.001), cyanosis (χ² = 12.91, *p* = 0.002), spasmodic cough(χ² = 212.07, *p* < 0.001), cockcrow-like echo(χ^2^ = 77.38, *p* < 0.001), cough duration prior to admission(H = 371.49, *p* < 0.001), lung crackles (χ² = 52.44, *p* < 0.001), and multilobar involvement (χ² = 28.08, *p* < 0.001). Specifically, the MP-only group experienced significantly more frequent fever than both the pertussis-only and dual-positive groups. Gastrointestinal symptoms and, cyanosis, Spasmodic Cough and cockcrow-like echo were more common in the pertussis-only group, while lung crackles and multilobar involvement were most frequent in the MP-only group. The duration of cough prior to hospital admission was shorter in the MP-only group compared to both the pertussis-only and dual infection groups (**Table 4**).

**Table 4:** Comparison of clinical manifestations and hematologic indices by positivity pattern.

Indicator Group	Pertussis-positive only (n=2982)	MP-positive only (n=3166)	Dual-positive (n=1036)	Total	Test statistic	*p-* value
Clinical manifestations[n(%)]						
Wheeze or shortness of breath	329 (40.47%)	382 (46.99%)	102 (12.55%)	813	χ^2^ = 4.24	0.120
Fever	725 (26.80%)^a^	1593 (58.89%)^b^	387 (14.31%)^c^	2705	χ^2^ = 442.36	<0.001
Gastrointestinal symptoms	794 (46.19%)^a^	660 (38.39%)^b^	265 (15.42%)^a^	1719	χ^2^ = 30.00	<0.001
Cyanosis	28 (68.29%)^a^	8 (19.51%)^b^	5 (12.20%)^ab^	41	χ^2^ = 12.91	0.002
Dyspnea	32 (41.56%)	34 (44.15%)	11 (14.29%)	77	χ^2^ = 0.001	0.999
Spasmodic Cough	305 (70.28%)^a^	48 (11.06%)^b^	81 (18.66%)^a^	434	χ^2^ = 212.07	<0.001
Cockcrow- Like Echo	140 (67.31%)^a^	31 (14.90%)^b^	37 (17.79%)^a^	208	χ^2^ = 77.38	<0.001
Apnea	4 (66.67%)	2 (33.33%)	0 (0%)	6	–	0.532
Cough duration before diagnosis(days)[M ± IQR]	15 ± 20^a^	10 ± 16^b^	15 ± 20^a^	14 ± 23	H=371.49	<0.001
Lung crackles	85 (25.00%)^a^	213 (62.65%)^b^	42 (12.35%)^a^	340	χ^2^ = 52.44	<0.001
Multilobar involvement	1251 (39.52%)^a^	1503 (47.47%)^b^	412 (13.01%)^a^	3166	χ^2^ = 28.08	<0.001
Hematologic indices[M ± IQR]						
Lymphocyte count	3.77 ± 2.43^a^	3.23 ± 2.00^b^	3.45 ± 2.03^c^	3.47 ± 2.19	H= 178.03	<0.001
Neutrophil count	3.83 ± 3.02^a^	4.50 ± 3.35^b^	4.04 ± 3.15^c^	4.19 ± 3.20	H= 119.45	<0.001
White blood cell count	8.89 ± 4.47	8.95 ± 4.21	8.73 ± 4.09	8.89 ± 4.31	H = 3.07	0.215
Platelet count	325.00 ± 113.00	330.50 ± 127.00	326.00 ± 110.00	328.00 ± 120.00	H = 0.59	0.743
C-reactive protein	0.50 ± 2.36^a^	1.80 ± 5.50^b^	0.50 ± 2.63^c^	0.50 ± 2.84	H = 369.8	<0.001

Superscript letters indicate subsets of positive types; same letters indicate no significant difference, different letters imply a significant difference (P<0.05).

For hematologic indices, the three groups showed significant differences in terms of lymphocyte count (H = 178.03, *p* < 0.001), neutrophil count (H = 119.45, *p* < 0.001), and C-reactive protein (H = 369.8, *p* < 0.001). Lymphocyte counts were significantly higher in the pertussis-only group than in the MP-only and dual-positive groups, whereas neutrophil counts and C-reactive protein were significantly higher in the MP-only group (**Table 4**).

### 3.6 Relationship between positivity pattern and co-detected pathogens

#### 3.6.1 Distribution of co-detected pathogens by positivity pattern

Significant differences were observed across positivity patterns for influenza A/B viruses ((IAV/IBV) (χ² = 16.688, *p* < 0.001), *Legionella pneumophila(L. pneumophila)* (χ² = 12.715, *p* = 0.002), *Streptococcus pneumoniae (S. pneumoniae)* (χ² = 11.872, *p* = 0.003), and *Klebsiella pneumoniae (K. pneumoniae)* (χ² = 7.284, *p* = 0.009). Posthoc pairwise comparisons revealed a lower IAV/IBV positivity in the pertussis-only group than in the MP-only and dual-positive groups; higher *L. pneumophila* positivity in the pertussis-only group than in the MP-only group; higher *S. pneumoniae* positivity in the MP-only group than in the pertussis-only and dual-positive groups; lower *K. pneumoniae* positivity was in the MP-only group than in the dual-positive group (**Table 5**).

**Table 5:** Co-detected pathogens by positivity pattern.

Pathogen Group	Pertussis-positive only (*n* = 2982)	MP-positive only (*n* = 3166)	Dual-positive (*n* = 1036)	Total	Test statistic	*p-*value
Viruses						
IAV	30 (44.78%)	31 (46.27%)	6 (8.96%)	67	1.649	0.438
IBV	24 (43.64%)	21 (38.18%)	10 (18.18%)	55	1.040	0.594
HPIV-3	14 (50.00%)	13 (46.43%)	1 (3.57%)	28	2.818	0.244
HPIV-1	0 (0.00%)	1 (100.00%)	0 (0.00%)	1	1.598	1.000
IAV/IBV	17 (22.37%)^a^	38 (50.00%)^b^	21 (27.63%)^b^	76	16.688	<0.001
HPIVs	40 (36.70%)	59 (54.13%)	10 (9.17%)	109	5.271	0.072
HRV	151 (38.13%)	184 (46.46%)	61 (15.40%)	396	1.978	0.372
HAdV	86 (39.09%)	102 (46.36%)	32 (14.55%)	220	0.593	0.743
HBoV	4 (30.77%)	7 (53.85%)	2 (15.38%)	13	0.653	0.722
HMPV	16 (34.78%)	21 (45.65%)	9 (19.57%)	46	1.380	0.501
Bacteria						
*H. influenzae*	141 (36.81%)	179 (46.74%)	63 (16.45%)	383	3.954	0.138
*L. pneumophila*	25 (67.57%)^a^	6 (16.22%)^b^	6 (16.22%)^ab^	37	12.715	0.002
*S. pneumoniae*	95 (35.45%)^a^	145 (54.10%)^b^	28 (10.45%)^a^	268	11.872	0.003
*S. aureus*	13 (41.94%)	14 (45.16%)	4 (12.90%)	31	0.060	0.971
*P. aeruginosa*	4 (40.00%)	5 (50.00%)	1 (10.00%)	10	0.178	1.000
*K. pneumoniae*	3 (50.00%)^ab^	0 (0.00%)^b^	3 (50.00%)^a^	6	7.284	0.009
Atypical						
*C. pneumoniae*	2 (15.38%)	7 (53.85%)	4 (30.77%)	13	4.837	0.089

Superscript letters indicate subsets of positive types; same letters indicate no significant difference, different letters imply a significant difference (P<0.05).

We analyzed the differences in the distribution of co-detected pathogens among the three positivity patterns. Chi-square tests unveiled significant differences for IAV/IBV (χ² = 16.688, *p* < 0.001), *L. pneumophila* (χ² = 12.715, *p* = 0.002), *S. pneumoniae* (χ² = 11.872, *p* = 0.003), and *K. pneumoniae* (χ² = 7.284, *p* = 0.009). Pairwise comparisons indicated a significantly lower IAV/IBV positivity in the pertussis-only group than in the MP-only and dual-positive groups; significantly higher *L. pneumophila* positivity in the pertussis-only group than in the MP-only group; significantly higher in *S. pneumoniae* positivity the MP-only group than in the pertussis-only and dual-positive groups; significantly lower *K. pneumoniae* positivity in the MP-only group than in the dual-positive group (**Table 5**).

#### 3.6.2 Combination characteristics of co-detected pathogens by positivity pattern

**Figure 1:**
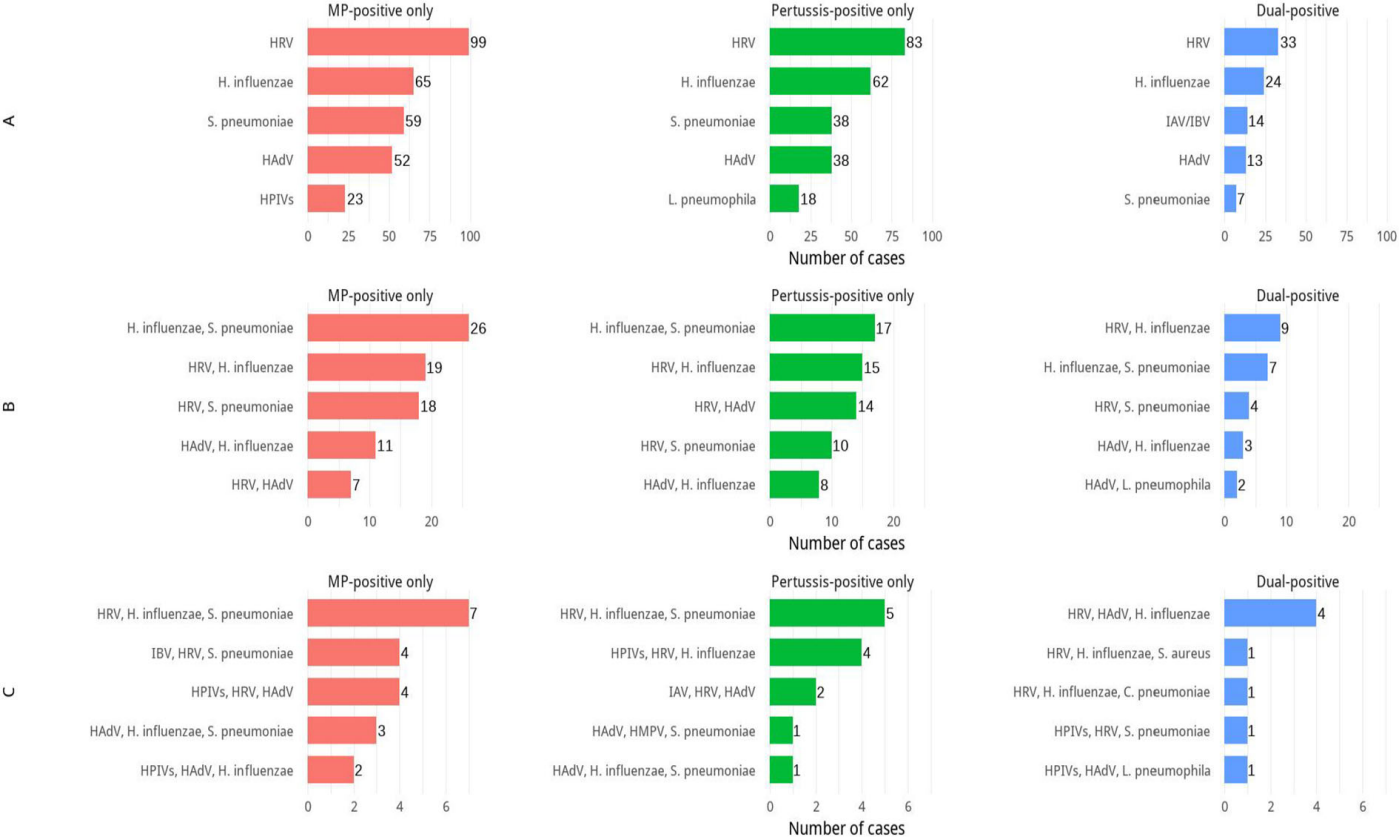
Top 5 Combinations of co-detected pathogens across different positivity patterns. **(A)** Five most frequent combinations involving one additional pathogen within each positivity type; **(B)** five most frequent combinations involving two additional pathogens within each positivity type; **(C)** five most frequent combinations involving three additional pathogens within each positivity type.

In addition to the distributional differences noted above, we summarized the frequency and common constellations of codetections. Of 7,184 specimens, 1,224 (15.65%) had at least one additional respiratory pathogen detected. Among these, single-pathogen co-detections were most frequent (n=799, 65.28%), and across all three positivity patterns, the leading copathogens were human rhinovirus (HRV) and *Haemophilus influenzae* (*H. influenzae*). Two-pathogen codetections occurred in 328 cases (26.80%), most commonly *H. influenzae* with *S. pneumoniae* or with HRV. Codetections involving three or more pathogens were identified in 97 cases (7.92%); across positivity patterns, the most frequent combinations were *H. influenzae* plus HRV together with *S. pneumoniae* or human adenovirus (HAdV)(**Figure 1**).

## 4 Discussion

This study retrospectively analyzed children who tested positive for pertussis, MP, or both over the past five years in our hospital. Three positivity patterns were compared in terms of demographic characteristics, seasonal distribution, clinical manifestations, laboratory findings, and co-detected pathogens. The data provide clinical evidence for better understanding of the interplay and distinct characteristics of pertussis and MP infections in children.

We observed significant sex differences across positivity patterns. Prior work indicates that sex determines the susceptibility to infectious diseases: females often mount stronger humoral and cellular immune responses to bacterial, viral, and fungal pathogens, whereas males tend to have higher risk and more severe courses; these differences likely reflect contributions from genetics, epigenetic regulation, and sex hormones (Gay et al., 2021). In our research, the pertussis-only group included a higher proportion of boys, consistent with previous reports (Ning et al., 2018). By contrast, a meta-analysis revealed a higher pertussis incidence among females in certain age strata, most notably in infancy, which suggests that sex effects are modified by age and immune status (Peer et al., 2020). For MP, we detected no sex-based difference, in line with domestic studies (Jiang et al., 2024). Notably, girls were over-represented in the dual-positive group, but systematic evidence on sex differences in co-infection remains limited. Previous studies suggest that sex can influence the strength of both innate and adaptive immune responses as well as vaccine efficacy, and that these effects may vary with age and immune status. Therefore, in the context of co-infection with multiple pathogens, sex differences could lead to different susceptibility patterns or pathogen detection profiles (Klein and Flanagan, 2016). Given the limited direct evidence on sex differences in co-infections, further studies with larger sample sizes and age-stratified cohorts are needed to confirm these findings. These findings may have implications for clinical decision-making, particularly in the diagnosis and treatment of co-infections.

We also observed notable age-related differences across positivity patterns. Infants are intrinsically vulnerable to infection and severe outcomes given their still-maturing innate and adaptive immunity (Pieren et al., 2022). With the increase in age, immune function improves, but close contact in childcare and school environments sustains higher exposure for preschool- and school-age children (Andrup et al., 2024). Historical reports on pertussis emphasize infancy (Huang et al., 2017; Skoff et al., 2019). By contrast, the proportion of pertussis-only cases in our study was the highest at 6 to <12 years, consistent with the upward shift in age at onset described during the pertussis resurgence—extending to older children, adolescents, and even adults (MacIntyre et al., 2024). This suggests that, over time, the immune protection provided by the pertussis vaccine gradually diminishes, especially several years after vaccination, increasing children’s susceptibility to re-infection (Hu et al., 2024). Notably, with adjustments to the immunization schedule, particularly the introduction of booster doses during school years, future efforts may improve immune protection in children and adolescents, potentially mitigating the increasing susceptibility observed in these age groups. Furthermore, changes in the immunization schedule may impact the epidemiological characteristics of pertussis and the clinical presentation of vaccinated individuals with post-vaccination infections, posing new challenges for clinical diagnosis and case surveillance. And advances in diagnostic methods and clinical awareness have improved the recognition of atypical cases (Hua et al., 2024). Collectively, these factors might have contributed to the upward shift in the age distribution of pertussis.

For MP infection, prior studies have mainly involved school-age children (Qu et al., 2018; Kutty et al., 2019; Kong et al., 2024). Our results also confirm this clinical pattern: MP-only cases were predominantly observed in children aged to <12 years, and this finding may be related to the frequent close contact in enclosed settings, such as kindergartens and schools, and the higher risk of droplet transmission in these environments. In addition, we noted a relatively high proportion in the 2 to <6 years group, and approximately 12% in the 3 months to <2 years group, consistent with a trend toward younger age at onset of MP infection. Importantly, dual-positive cases were mainly concentrated in the 6 to <12-year group, which suggests that this age band may have become a high-risk population for pertussis–MP co-infection and further complicating clinical differentiation. Given that both infections can present with nonspecific features—paroxysmal cough, wheeze, and fever—early clinical distinction is difficult (Hanna and Samies, 2025; Wolski et al., 2025), and age-based diagnostic heuristics no longer fit the current epidemiology. Moreover, although macrolides remain the first-line treatment for both pathogens, resistance to it is substantial. In some patients, second-line agents are an unavoidable choice, yet the regimens differ markedly. Delayed etiologic identification may result in the postponement or inappropriate selection of treatment, which adversely affects outcomes (Guo et al., 2022; Ivaska L et al., 2022). In view of our previous clinical studies (Han et al., 2016; Meng et al., 2017; Yan et al., 2024), we recommend routinely including pertussis in testing for persistent cough or acute irritative cough and maintaining vigilance for MP. Against a backdrop of shifting epidemiologic trends and overlapping symptoms, broader adoption of multiplex PCR can improve pathogen identification, guide rational antimicrobial use, and standardize management.

Pertussis-only cases were mainly concentrated in winter and spring, which is inconsistent with pre-COVID reports of summer–autumn peaks in Northern China (Han et al., 2016; Liu et al., 2017; Zhang et al., 2024). This change suggests that COVID-19 control measures might have affected the seasonal epidemiology of pertussis in northern regions (Mengyang et al., 2024), with potential reasons including alterations in population behavior during the pandemic, adjustments in the circulation patterns of seasonal respiratory viruses, and weakening of the population immunity barrier to pertussis. For Mycoplasma pneumoniae (MP), MP-only cases were mainly concentrated in summer and autumn, showing clear seasonality consistent with previous reports (Zhang J et al., 2025), although some studies have noted autumn–winter peaks (Wang et al., 2022), MP circulation was also disrupted by the pandemic: in Wuhan, peaks most often occurred in summer before the pandemic; during the pandemic, annual peaks shifted initially to spring and then to summer and then shifted to late autumn in the post-pandemic period (Qiu et al., 2024). These studies suggest that MP seasonality may be jointly influenced by regional climate, pandemic control measures, and patterns of human contact. In addition, dual-positive cases were mainly concentrated in spring and summer,which possibly reflects the partial overlap of pertussis and MP peaks during this period. Further analysis showed that differences among the three positivity patterns were most pronounced in summer. Altogether, these findings reveal the complexity of pathogen infection and provide ideas for season-specific pathogen screening strategies to improve the timeliness and accuracy of clinical diagnosis.

Our study indicated that clinical manifestations and laboratory indices differed across positivity patterns. Children in the MP-only group were more likely to have fever, lung crackles, and multilobar involvement, accompanied with higher neutrophil counts and C-reactive protein, which reflects typical acute infection/pneumonia profile; these findings are largely consistent with prior reports and suggest a stronger systemic inflammatory response following MP infection (Defilippi et al., 2008). In contrast, children in the pertussis-only group exhibited lower fever rates, but had higher incidences of gastrointestinal symptoms, cyanosis, paroxysmal coughing, and whooping sounds, along with relatively higher lymphocyte counts. Paroxysmal coughing and whooping sounds are characteristic symptoms of pertussis and have high clinical specificity (Mi et al., 2024).Furthermore, we found that the clinical presentation of pertussis has changed, particularly with a reduced incidence of fever, where other atypical symptoms (such as gastrointestinal symptoms and cyanosis) were also more common in the pertussis-only group. Previous studies have focused on atypical presentations of pertussis in infants, such as the absence of fever, and the presence of respiratory pauses or feeding difficulties (Decker and Edwards, 2021; Leontari et al., 2025). However, with changes in vaccination coverage, atypical presentations have been widely reported in older children, adolescents, and even adults, commonly including persistent cough and mild respiratory symptoms, which may be misdiagnosed as ordinary respiratory infections or asthma (Mi et al., 2024). This suggests that clinicians need to consider the possibility of atypical pertussis across different age groups. Further analysis revealed that the duration of cough before diagnosis in the pertussis-only group was significantly longer than in the MP-only and dual-positive groups, consistent with the typical characteristics of pertussis. Pertussis typically presents with gradually worsening cough that persists longer (Decker and Edwards, 2021), whereas MP infection initially manifests as pharyngitis, hoarseness, and fever, with persistent day-and-night coughing being a hallmark of infection spreading to the lower respiratory tract (Kashyap and Sarkar, 2010). The duration of cough in the dual-positive group was between that of the two single-positive groups. Case reports and observations have noted that dual infection may lead to more complex or aggravated clinical symptoms (Zouari et al., 2012). Assessing cough duration prior to confirming a diagnosis of pertussis or Mycoplasma infection is crucial, as prolonged cough may lead to overlapping symptoms, making diagnosis more difficult, particularly in populations with incomplete or weakened vaccination. Cough duration can aid in improving early diagnostic accuracy and avoiding confusion with other infections. Additionally, children in the pertussis-only group had higher lymphocyte counts, which is consistent with the typical immune response triggered by pertussis infection (Huang et al., 2024). This suggests that pertussis infection is usually accompanied by a significant immune response, which can serve as a useful reference for clinical diagnosis. In addition, in the dual-positive group, the proportions of fever and pulmonary signs were lower, and hematologic indices were intermediate between the two single-positive groups. This pattern suggests that immune responses may be influenced by dual pathogens, which led to atypical inflammatory activation and more complex clinical manifestations and underscore the importance of multiplex pathogen testing.

Differences were also observed in the spectrum of co-detected pathogens across positivity patterns. IAV/IBV and *S. pneumoniae* were more frequently detected in the MP-only group, *L. pneumophila* in the pertussis-only group, and *K. pneumoniae* in the dual-positive group. These differences suggest that the immune response mechanisms of different pathogens vary in their effects on the host immune system. Specifically, MP infection activates the immune system, promoting the recruitment and activation of neutrophils, which damages the airway epithelium and weakens the host’s immune defense, thus increasing susceptibility to influenza virus and *S. pneumoniae* (Chen et al., 2016). Previous studies have shown that respiratory viral co-infections with MP are common in children and are associated with more severe symptoms, longer hospital stays, and more complications (Yu et al., 2024). Multi-omics analysis also indicates that patients with MP and viral co-infections have significant pro-inflammatory metabolic changes and elevated chemokine levels in bronchoalveolar lavage fluid, suggesting a stronger inflammatory environment in these patients (Li et al., 2025). In contrast, *Bordetella pertussis* may suppress neutrophil function and immune cell recruitment through immune evasion mechanisms, leading to a weakened immune response against intracellular pathogens such as *L. pneumophila* (Andreasen and Carbonetti, 2008). This immune evasion in co-infected patients likely exacerbates disease severity. A retrospective study noted that nearly 40% of B. pertussis infections involved co-detected pathogens, which correlated with higher inflammatory markers (e.g., CRP, ferritin) and lower oxygen saturation, indicating worsened clinical outcomes (Matache Vasilache et al., 2025). Other evidence suggests that B. pertussis may facilitate co-detection of additional pathogens or contribute to complex co-infection patterns, potentially leading to more severe lower respiratory tract infections (Muloiwa et al., 2020).

Based on the distinct demographic, seasonal, clinical, and laboratory patterns observed, clinicians should consider simultaneous multiplex PCR testing for pertussis and MP in children with overlapping respiratory symptoms, particularly during peak seasons. Selected laboratory markers may provide additional support for early differentiation and help guide more targeted clinical management.

This study encountered several limitations. First, it is a single-center retrospective analysis, which may introduce selection bias and limit the generalizability of the findings. The results may not fully reflect the diversity of patient populations in other healthcare settings or regions. Second, variations across different postpandemic periods might have been influenced by public health control measures, which potentially affect the regularity of pathogen detection. Third, as the study primarily focused on co-detection rates, it did not further assess whether the pathogens represented true clinical infections, which limited the interpretation of the findings.

Vased on a large cohort of clinical data, this work confirmed that pertussis and MP infections showed substantial overlap in affected populations, with school-aged children emerging as a common high-risk group. Complex seasonal distribution patterns were observed, and although differences in sex distribution were recorded, their clinical significance remains unclear. Moreover, the clinical manifestations of both infections showed a high similarity. Apart from molecular pathogen detection, no reliable clinical indicators were identified for rapid differentiation, which poses challenges for diagnosis. Overall, these findings highlight the substantial clinical overlap and diagnostic challenges of pertussis and MP infections in children and underscore the importance of accurate etiologic identification in the context of changing epidemiology.

## Data Availability

The raw data supporting the conclusions of this article will be made available by the authors, without undue reservation.
